# Polymer Film Supported Bimetallic Au–Ag Catalysts for Electrocatalytic Oxidation of Ammonia Borane in Alkaline Media

**DOI:** 10.1007/s40820-016-0095-3

**Published:** 2016-06-14

**Authors:** Şükriye Ulubay Karabiberoğlu, Çağrı Ceylan Koçak, Süleyman Koçak, Zekerya Dursun

**Affiliations:** 1 Department of Chemistry, Science Faculty, Ege University, 35100 Bornova, Izmir, Turkey; 2 Occupational Health and Safety Department, Bergama Vocational School, Dokuz Eylul University, Izmir, Turkey; 3 Department of Chemistry, Science and Art Faculty, Celal Bayar University, 45040 Manisa, Turkey

**Keywords:** Au–Ag bimetallic nanoparticles, Ammonia borane, Electrocatalyst, Electropolymerization, Alkaline media

## Abstract

**Abstract:**

Ammonia borane is widely used in most areas including fuel cell applications. The present paper describes electrochemical behavior of ammonia borane in alkaline media on the poly(*p*-aminophenol) film modified with Au and Ag bimetallic nanoparticles. The glassy carbon electrode was firstly covered with polymeric film electrochemically and then, Au, Ag, and Au–Ag nanoparticles were deposited on the polymeric film, respectively. The surface morphology and chemical composition of these electrodes were examined by scanning electron microscopy, transmission electron microscopy, electrochemical impedance spectroscopy, X-ray diffraction, and X-ray photoelectron spectroscopy. It was found that alloyed Au–Ag bimetallic nanoparticles are formed. Electrochemical measurements indicate that the developed electrode modified by Au–Ag bimetallic nanoparticles exhibit the highest electrocatalytic activity for ammonia borane oxidation in alkaline media. The rotating disk electrode voltammetry demonstrates that the developed electrode can catalyze almost six-electron oxidation pathway of ammonia borane. Our results may be attractive for anode materials of ammonia borane fuel cells under alkaline conditions.

**Graphical Abstract:**

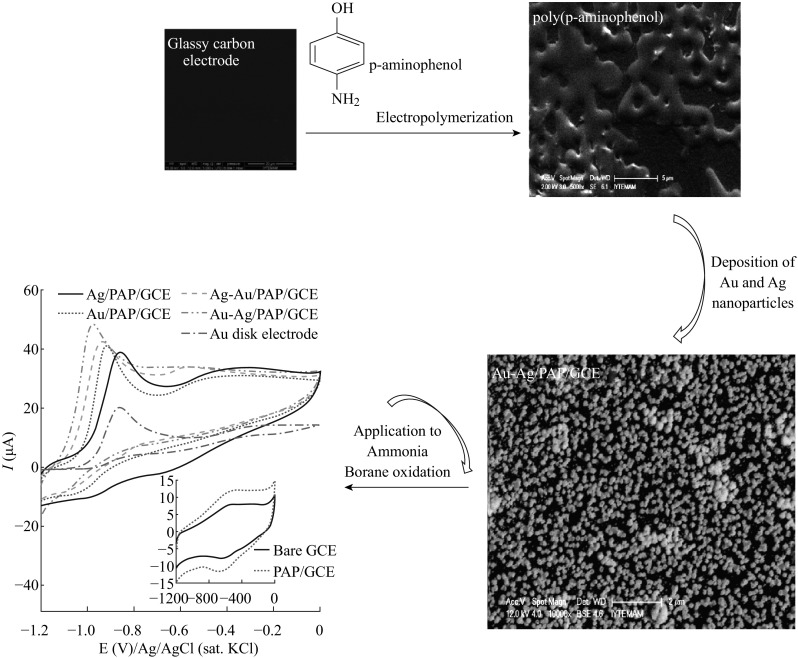

## Introduction

Ammonia borane (or borazane, NH_3_BH_3,)_ is a crystalline, white colored, non-toxic and stable chemical, which is an alternative source for hydrogen storage due to its high hydrogen content (19.5 wt%) and appropriate temperature for H_2_ release. It can be easily transported and dissolved in polar solvents such as water and methanol [1–3]. Ammonia borane (AB) has been used in fuel cells in two ways: one is H_2_ formed hydrolysis of AB source [4–6] and another is AB direct electrooxidation [7]. The hydrolysis of AB is unwanted in AB electrooxidation reaction since the H_2_ evolution in the system not only reduces the energy density of fuel cell but also leads to the safety problems. The hydrolysis reaction of AB has been observed in the presence of some common catalysts such as Co [8, 9], Pd [10], Ru [11], Ni [12], Co-Pt [13], NiCo-Pt [14], etc. Obviously, to avoid the hydrolysis of AB, these metal catalysts should not be used in ammonia borane electrooxidation reaction directly.

The direct electrooxidation of AB is assumed as chemical or electrochemical reaction, which is initiated with the dissociation of AB in alkaline solution to form hydroxyl-borohydride (BH_3_(OH)^−^) and ammonia (NH_3_) (Eq. 1). The BH_3_(OH)^−^ intermediate is then oxidized to form B(OH)_4_^−^ with a yielding of total six electrons related to the maximal efficiency process [15, 16] (Eq. 2):

1 $${\text{NH}}_{3} {\text{BH}}_{{3_{{\left( {\text{aq}} \right)}} }} + {\text{OH}}_{{\left( {\text{aq}} \right)}}^{ - } \rightleftarrows {\text{NH}}_{{3_{{\left( {\text{aq}} \right)}} }} + {\text{BH}}_{3} ({\text{OH}})_{{\left( {\text{aq}} \right)}}^{ - }$$

2 $${\text{BH}}_{3} ({\text{OH}})_{{\left( {\text{aq}} \right)}}^{ - } + 6{\text{OH}}_{{\left( {\text{aq}} \right)}}^{ - } \rightleftarrows {\text{B}}({\text{OH}})_{{4_{{\left( {\text{aq}} \right)}} }}^{ - } + 3{\text{H}}_{2} {\text{O}} + 6{\text{e}}^{ - }.$$

The electrooxidation of AB may proceed through different paths as multielectron process, generally depending on the electrode material and pH of the supporting electrolyte. In order to hinder hydrolysis reaction of AB, the electrode material is generally inactive for hydrolysis by increasing the pH of supporting electrolyte. Au-based electrodes are good catalysts for direct electrochemical oxidation of BH_3_(OH)^−^ and show poor catalytic activity with respect to its hydrolysis over a certain concentration range [16–18]. The direct electrochemical oxidation of AB (Eqs. 1, 2) has been studied on Au microdisk and Au disk electrodes in alkaline solutions [17, 18]. In another study, amorphous core of Fe in Fe–Pt core–shell nanoparticles showed high catalytic activity towards AB oxidation [19].

Gold nanoparticles are remarkable examples of nanoscaled electrocatalysts with new properties that have been used in fuel cells [20–23], sensors [24–26], as well as many other applications due to their small dimensional size, good stability, and excellent catalytic activity. Silver nanoparticles are also widely used in electrochemistry as electrode materials due to their high catalytic activity, novel optical property, and low cost compared to other noble metals [27–31]. In our previous study, Ag nanoparticles were synthesized on poly(thiophene)-modified glassy carbon electrode (GCE) surface using cyclic voltammetry (CV) and then applied to the electrocatalytic caffeic acid oxidation [32].

Bimetallic nanoparticle-modified electrodes have been studied extensively due to their improved catalytic performance owing to their synergistic and electronic effects [33–37]. Synthesis of bimetallic particles can be carried out through various methods, such as electroless plating [37], surface reaction [38], seeding-mediated synthesis [39], self-assembly, and electrochemical methods [40, 41]. Among them, electrochemical synthesis is an easy approach in preparing bimetallic surfaces by controlling the impurities, particle sizes, and the amount of modifier components. Metal nanoparticles need a supporting material such as carbon nanotubes, graphene, or conducting polymers in order to maintain their stability and physical properties [42, 43].

Au–Ag bimetallic catalysts have concerned much attention in recent years owing to their electronic, optical, and catalytic properties which differ from those of individual Au and Ag mono metals. Au–Ag bimetallic catalysts have been widely used for catalytic applications [44]. Reduced graphene oxide supported Au–Ag bimetallic catalyst was found to have higher electrocatalytic activity for glucose compare Au and Ag monometal catalysts [45]. In another study, grapheme was used for supporting of sonochemical synthesis of Au–Ag bimetallic nanoparticles to improve the catalytic activity of graphene oxide for investigation of 4-nitrophenol [46]. Au–Ag alloy catalyst was also supported on mesoporous aluminosilicate for use in low-temperature CO oxidation with high stability [47]. In addition, Au–Ag bimetallic nanoparticle-modified glassy carbon electrode was used as H_2_O_2_ sensor, which was directly deposited on GCE surface by CV [48]. Another sensing application is that pyrene determination on Au–Ag bimetallic nanoparticle-modified poly(pyrrole) film GCE in which Au and Ag nanoparticles were successfully synthesized by co-precipitation method [49].

To the best of our knowledge, the present study is the first report for the electrochemical oxidation of AB at electrochemical fabrication of Au–Ag nanoalloy on PAP surface. The poly(*p*-aminophenol) film glassy carbon electrodes (PAP/GCE) were modified with Au–Ag bimetallic nanoparticles by electrochemical techniques for catalytic oxidation of AB in alkaline solution. The morphological, electrical, and chemical properties of all electrodes were investigated. The electron-transfer number during ammonia borane oxidation was also calculated by using rotating disk electrode and chronoamperometry studies.

## Experimental

### Reagents

AB was purchased from Alfa Aesar. Standard solutions of AB (0.1 mol L^−1^) were freshly prepared by dissolving the required amount of reagent in ultrapure water. NaOH was used as a supporting electrolyte obtained from Raidel De Haen. The *p*-aminophenol was obtained from Fluka and used without further purification. Sodium dodecylsulfate (SDS), HClO_4_, AgNO_3_, and HNO_3_ were of analytical-reagent grade and was supplied from Sigma, Carlo Erba, and Merck, respectively. Chloroauric acid solution was prepared by dissolving the Au wire (99.999 % in purity, Tanaka Kikinzoku Kogyo Co., Ltd.) in a mixture of concentrated HNO_3_:HCl (volume ratio of 1:3). All of the solutions were prepared by using ultrapure water with minimum resistance of 18 MΩ cm^−2^ from Milli-Q system. All experiments were carried out at ambient temperature and under highly pure nitrogen flow over the solution during electrochemical experiments.

### Instruments

Voltammetric measurements were carried out using BAS100B/W Electrochemical Analyzer and Autolab 302N Voltammetric Analyzer equipped with three electrode system consisted of the as-synthesized working electrodes (GCE, PAP/GCE, Ag/PAP/GCE, Au/PAP/GCE, and Au–Ag/PAP/GCE), an auxiliary electrode (Pt wire) and reference electrode (Ag/AgCl (sat. KCl)). Glassy carbon electrode (with a diameter of 3 mm, 0.0707 cm^2^ geometric area) was purchased from BASi. Cyclic voltammetry, chronoamperometry modes, and rotating disk electrode were utilized throughout the studies.

The morphology and size of synthesized samples were determined by employing Philips XL 30 SFEG scanning electron microscopy (SEM) and Tecnai G2 F20 S-TWIN transmission electron microscopy (TEM). The elemental analysis was performed with an energy dispersive X-ray (EDX, Oxford). The X-ray photoelectron spectroscopy (XPS) measurements of the Au–Ag nanoparticle-modified PAP/GCE was carried out with a Thermo K-Alpha-Monochromated high-performance XPS spectrometer. The surface layer kinetics of modified electrodes were evaluated on Autolab 302 N electrochemical impedance spectroscopy (EIS). X-ray diffraction(XRD) patterns of the Au–Ag/PAP film electrode were obtained by X-ray diffraction analyses using PANALYTICAL Empyrean diffractometer with Cu-K-Alpha1 radiation (15406 Ao; 40 kV, 40 mA) and the samples were scanned from 5 to 90 2*θ* in step sizes of 00130 and scan step time 14,892 s.

### Preparation of Au–Ag Nanoparticle-Modified Poly(*p*-aminophenol) Film GCE

Prior to modification, GCE was activated by polishing with different grades of Al_2_O_3_ slurry (0.05–3 micron) on a synthetic cloth, and then rinsed with pure water and ultrasonicated in ultrapure water and ethanol for 3 min, successively. The PAP film was prepared according to previous studies [50]. The electrochemical polymerization of *p*-aminophenol was carried out in 5 mmol L^−1^ SDS and 5 mmol L^−1^ *p*-aminophenol containing 0.5 mol L^−1^ HClO_4_ solution by cycling the potential from −0.5 to 2.0 V versus Ag/AgCl (sat. KCl) with scan rate of 100 mV s^−1^. The obtained electrode was ready for use after the final wash with ultrapure water and denoted as PAP/GCE.

Ag nanoparticles were deposited on the PAP/GCE using CV according to our previous study [43]. Briefly, the prepared PAP/GCE electrode was immersed in 1 mmol L^−1^ AgNO_3_ and 0.1 mol L^−1^ HNO_3_ solution. The potential was scanned between 0.3 and −0.9 V by consecutive CVs with 50 mV s^−1^ scan rate for 10 cycles. The obtained metal nanoparticle-modified poly(*p*-aminophenol) film electrodes were ready to use after the final wash with ultrapure water and denoted as Ag/PAP/GCE. Deposition of gold nanoparticles on Ag/PAP/GCE was carried out in HAuCl_4_ and 0.1 mol L^−1^ HCl solution in the potential range between 0.1 to −0.9 V with 50 mV s^−1^ scan rate for 10 cycles. The obtained bimetallic-modified electrode was ready to use after the final wash with ultrapure water and denoted as Au–Ag/PAP/GCE.

## Results and Discussion

### Characterization of Electrodes

**Fig. 1:**
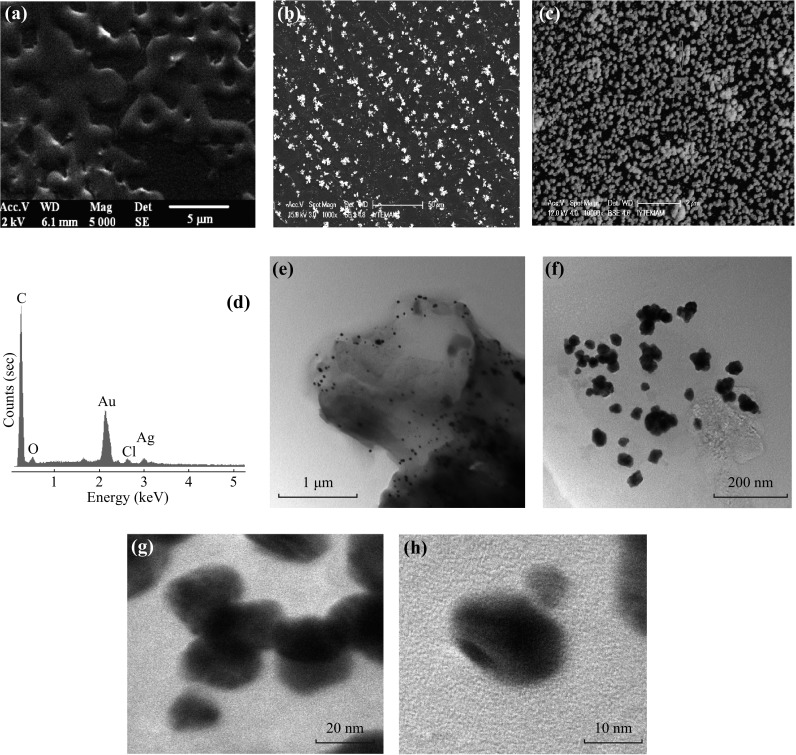
SEM images of **a** PAP/GCE, **b** Ag/PAP/GCE, and **c** Au–Ag/PAP/GCE, **d** EDX image of Au–Ag/PAP/GCE. **e**, **f** TEM and **g**, **h** HRTEM images of Au–Ag bimentallic nanoparticles on PAP/GCE film surface

The surface morphology of the bare GCE and modified electrodes were characterized by SEM and TEM. As it is seen in Fig. 1a, porous PAP film appears and shows almost homogenous distribution on the GCE surface which is obviously different from GCE image. On the PAP surface Ag nanoparticles with an average size of 80 nm are formed by cyclic voltammetry from SEM image of Ag/PAP/GCE (Fig. 1b). The particles were distributed uniformly on the PAP/GC working electrode. Figure 1c shows the SEM image of the PAP/GCE surface with Au–Ag bimentallic nanoparticles. As can be seen, the bimetallic nanoparticles are regularly distributed on the surface after consecutive deposition of Au and Ag on PAP/GCE. The nanoparticles have round shape with an average size of 90 nm. The presence of Au and Ag on PAP surface was confirmed by EDX spectroscopy analysis (Fig. 1d). The corresponding EDX analysis for the selected arrow point and square area of SEM are shown in Fig. 1c. The weight percentage of elements found in these areas were 33.07 % for Au and 4.88 % for Ag (Fig. 1d). The size and spherical morphology of Au–Ag bimentallic nanoparticles on the polymer substrate were also observed by TEM characterizations (Fig. 1e–h). At smaller magnifications, one can see the particles are well separated and the size is in the range of 20–50 nm (Fig. 1e, f). From the high-resolution TEM (HRTEM) images, the Au–Ag particles are substantially uniform, indicating that electron density within the volume of the particle is homogeneous (Fig. 1g, h). The data are in supportive agreement with previous reports [51, 52].

**Fig. 2:**
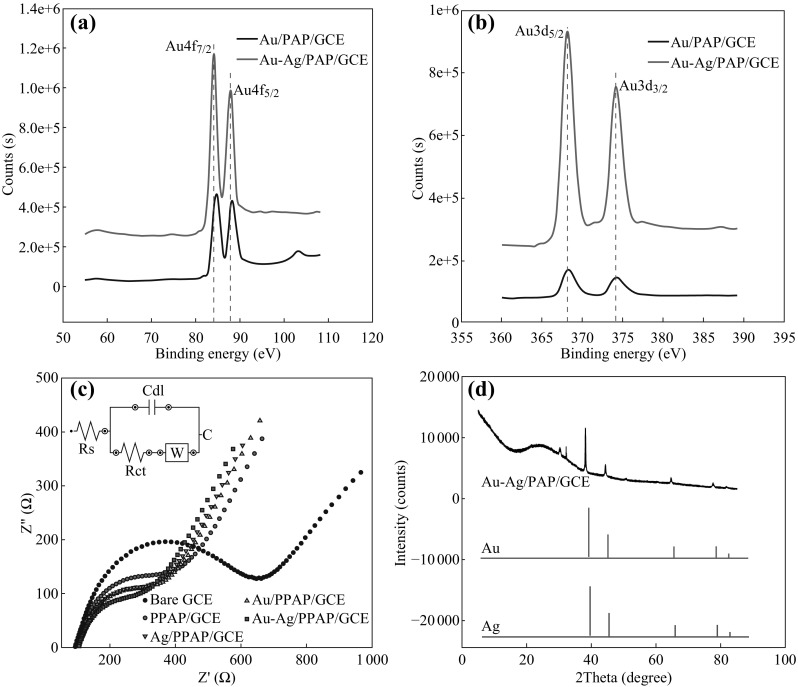
XPS spectra of **a** Au/PAP/GCE versus Au–Ag/PAP/GCE, **b** Ag/PAP/GCE versus Au–Ag/PAP/GCE. **c** The Nyquist plots of the bare GCE, PAP/GCE, Ag/PAP/GCE, Au/PAP/GCE, and Au–Ag/PAP/GCE. **d** XRD pattern of the Au–Ag/PAP/GCE electrode. The standard patterns of pure Au (ICSD 98-006-4701) and Ag (ICSD 98-006-4997) are attached for comparison

In order to characterize the chemical composition of the Au–Ag/PAP-modified glassy carbon electrode, Au4*f* and Ag3*d* photoemission core-level spectra were studied. Figure 2a, b shows the comparative core-level spectra for Au/PAP/GCE versus Au–Ag/PAP/GCE and Ag/PAP/GCE versus Au–Ag/PAP/GCE, respectively. The dashed lines indicate the positions of the bulk metal bands. The Au4*f* spectrum resolves into two spin–orbit components (Au4*f*_7/2_ and 4*f*_5/2_) on Au/PAP and Au–Ag/PAP electrodes (Fig. 2a). The peaks of Au4*f*_7/2_ are observed at 84.68 and 84.1 eV on Au/PAP electrodes, whereas peaks of Au4*f*_5/2_ are observed at 88.31 and 87.85 eV on Au–Ag/PAP, respectively. Au4*f* peaks at Au–Ag bimetallic surface are shifted towards lower binding energy (BE) by ~0.6 eV relatively to the bulk Au. The doublets are corresponding to the metallic Au [53]. The Ag3*d* signal of both Ag/PAP and Au–Ag/PAP electrode shows two peaks due to spine orbital splitting of the 3*d*_5/2_ and 3*d*_3/2_ states, as displayed in Fig. 2b. One of the intense doublet peaks, Ag3*d*_5/2_, were observed at 368.2 and 368.14 eV, and the other Ag3*d*_3/2_ peaks were observed at 374.3 and 374.15 eV on Ag/PAP and Au–Ag/PAP, respectively. The results could be attributed to the metallic Ag [54]. The evaluation of the Ag3*d* spectra of Au–Ag bimetallic surface reveals higher energy BE shift of ca. 0.1 eV related to the bulk metal value. The shifting of binding energy values indicates an alloyed formation with atomic level by mixing of Au and Ag. The 1.3:1.0 ratio of Au and Ag in Au–Ag/PAP electrode surface can be extracted from XPS data of 56.6 % for Au and 43.4 % for Ag atomic content.

Figure 2c shows EIS results of bare GCE, PAP/GCE, Au/PAP/GCE, Ag/PAP/GCE, and Au–Ag/PAP/GCE in the presence of 5 mmol L^−1^ [Fe(CN)_6_]^3−/4−^ containing 0.1 mol L^−1^ KCl solution at frequency range from 0.5 to 50.000 Hz. It can be seen that all electrodes represent a semi-circular and linear portion. The semi-circle corresponds to the charge transfer process through the GCE and polymer films at high frequencies, whereas the second one is due to the diffusion process in the low frequencies. The diameter of the semi-circle represents the magnitude of electron-transfer resistance at the electrode surface. The EIS data were fitted with an *R*_s_ (*C*(*R*_ct_ *W*)) equivalent circuit as given in Fig. 2c inset. This equivalent circuit consists of ohmic resistance (*R*_s_) of electrolyte solution, the double-layer capacitance (Cdl), and electron-transfer resistance (*R*_ct_) resulting from the diffusion of ions from bulk of the electrolyte to the interface and Warburg impedance (*W*). Large semi-circle as 428 Ω of *R*_ct_ value was obtained for bare GCE, indicating that there is a high electron-transfer resistance. However, after PAP film modification, a smaller semi-circle was obtained which meant low electron-transfer resistance. *R*_ct_ value obtained for PAP/GCE is 194 Ω which is higher than the metal nanoparticle-modified polymer film electrodes. The *R*_ct_ values of Au/PAP/GC and Ag/PAP/GC electrodes were found to be 157 and 154 Ω, respectively. The *R*_ct_ value of Au–Ag bimetallic surface is obviously smaller than other modified electrodes (126 Ω). This phenomenon proves the excellent electroconductibility of Ag–Au bimetallic surface. Moreover, these results show that Au–Ag/PAP film is successfully formed on the GCE surface.

The crystal structure of the Au–Ag/PAP-modified glassy carbon electrodes was also investigated and their XRD patterns are shown in Fig. 2d. As can be seen, five characteristic peaks of {111}, {200}, {220}, {311}, and {222} were, respectively, located at 38.28, 44.34, 64.54, 77.52, and 81.72, indicating an *fcc* Au–Ag alloy structure [55, 56]. Moreover, the XRD patterns of Au and Ag are completely overlapped since the lattice constants of Au (0.4080 nm) and Ag (0.4086 nm) are almost similar. Therefore, the Au–Ag alloy cannot be distinguished from the XRD powder patterns as monometallic phases [57]. Based on the experimental observations, the peak corresponding to the {111} plane is more intense than those to other planes, indicating that the {111} plane has the predominant orientation. It can be concluded that Au–Ag nanobimetallic structure has been successfully prepared on poly(*p*-aminophenol) surface by a simple electrode position using CV technique.

### Electrochemical Behavior of Ammonia Borane on Different Electrodes

**Fig. 3:**
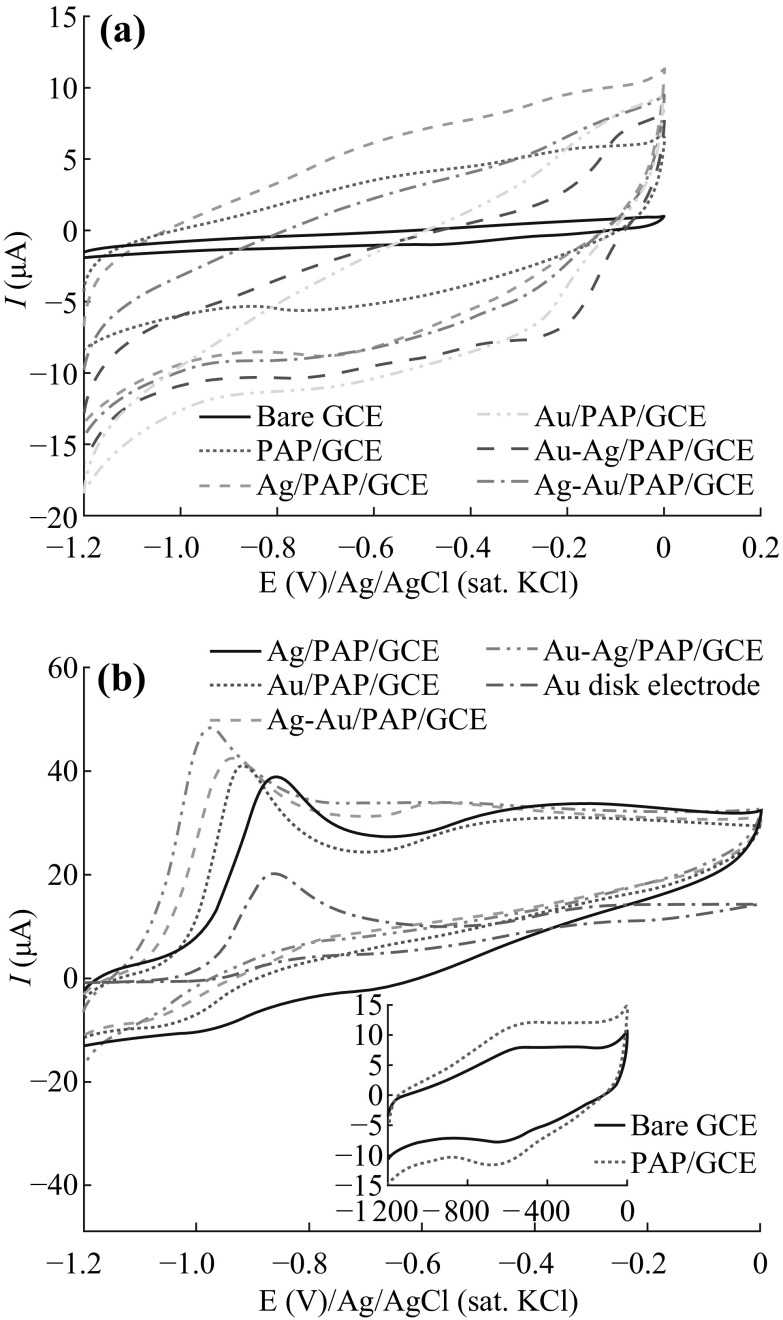
Cyclic voltammograms for bare GCE, PAP/GCE, Ag/PAP/GCE, Au/PAP/GCE, Au–Ag/PAP/GCE, and Ag–Au/PAP/GCE in 2.0 mol L^−1^ NaOH **a** absence of AB and **b** presence of 1.0 mmol L^−1^ AB, scan rate: 50 mV s^−1^

The electrochemical behaviors of various electrodes, such as bare GCE, PAP/GCE, Au disk electrode, Ag/PAP/GCE, Au/PAP/GCE, Ag–Au/PAP/GCE (firstly Au deposition, and then Ag deposition on Au/PAP/GCE), and Au–Ag/PAP/GCE (firstly Ag deposition, and then Au deposition on Ag/PAP/GCE) towards the AB oxidation were investigated in 2.0 M NaOH using CV technique. As can be seen from Fig. 3a, there isn’t any oxidation or reduction peak in either anodic or cathodic current—potential scan in the absence of AB at modified and bare electrodes. Modification of GCE with poly(*p*-aminophenol), Au and Ag nanoparticles resulted in the increment on background currents.

Figure 3b shows the cyclic voltammograms of the bare GCE, Au disk, and all modified electrodes in the 2.0 M NaOH solution containing 1.0 mM AB. Electrochemical behavior of AB on bare GCE and PAP/GC electrodes are given in the inset of Fig. 3b, in which there is no oxidation or reduction peaks observed in the presence of AB. This result shows that bare GCE and PAP/GCE surface have no catalytic affect towards AB electrooxidation. On the contrary, well-defined oxidation peaks were observed at ca. −0.863 V on Au disk, −0.917 V on Au_10cyc_/PAP, −0.865 V on Ag_10cyc_/PAP, −0.943 V on Ag_10cyc_–Au_10cyc_/PAP, and −0.978 V on Au_10cyc_–Ag_10cyc_/PAP-modified GC electrodes for 1.0 mmol L^−1^ AB in 2.0 mol L^−1^ NaOH solution. In addition, another small anodic peak as a shoulder was observed at about −0.40 V on these electrodes during the anodic way potential scans. Both oxidation peaks are related with the AB species that agree with the published work of Au nanoporous gold catalyst [16] and microelectrodes [17]. The first peak located at more negative potential may be assigned to the electrooxidation of BH_3_OH^−^ produced by chemical dissociation of BH_3_ from AB which reacts with OH^−^. The second small anodic peak located at more positive potential that can be associated with oxidation product of BH_3_OH^−^, without the implication of H_2_ electrooxidation.

The above CV results show that the Au–Ag nanoalloy exhibits much higher catalytic activity for the electrocatalytic oxidation of AB in terms of peak potential and peak current in comparison with other modified electrodes. The AB oxidation potential is more negative on Au–Ag surface than those observed on Au/PAP and Ag/PAP surfaces, respectively. The shifting of oxidation potential to more negative potentials could be attributed to the existence of bimetallic Au–Ag alloy particles on the polymer support that enhance the electrocatalytic oxidation kinetics of AB.

When compared the present results with reported papers [16–18], the electrochemical oxidation of AB was monitored at more negative potential on Au–Ag bimetallic surface than the published papers. The higher catalytic activity of Au–Ag/PAP/GCE can be explained by the synergetic effect of alloy bimetallic particles supported by XPS, XRD, and TEM studies.

**Fig. 4:**
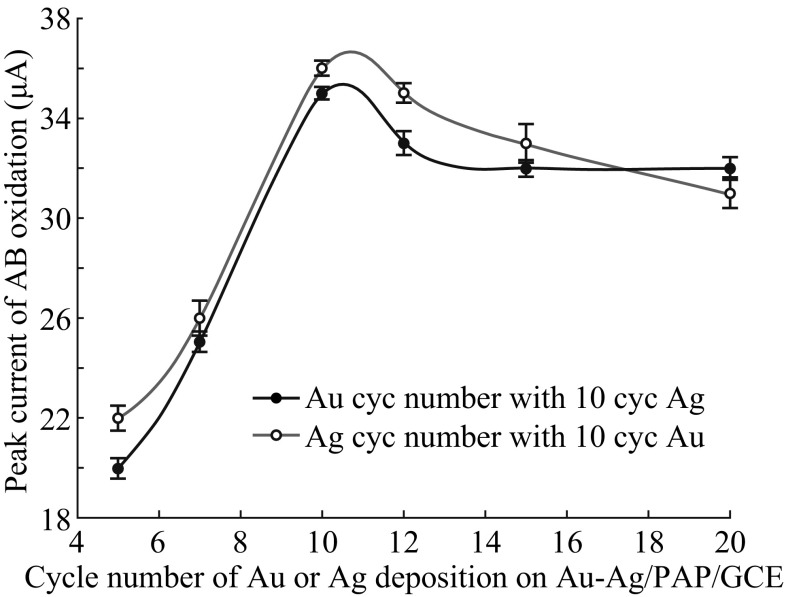
Dependence of Ag and Au modification cycle number on oxidation peak current of 1 mmol L^−1^ AB in 2 mol L^−1^NaOH

To prepare Au–Ag/PAP/GC electrode with improved electrocatalytic activity towards AB oxidation, a series of parameters were optimized, such as NaOH concentration, *p*-aminophenol concentration, and polymerization cycle number. After optimization studies, the optimum parameters were evaluated as follows: 2.0 M NaOH, 5.0 mM *p*-aminophenol concentration, and 35 cycles for polymerization. The PAP/GC electrodes were prepared with different coverages by changing the cycle numbers of Au and Ag deposition (Fig. 4) in order to obtain the best composition of Au–Ag on PAP surface for AB oxidation. The overall results prove that bimetallic nanoparticle-modified PAP/GC electrodes offer a satisfactory enhancement in peak characteristics of AB oxidation. The desirable change in oxidation peak current was observed for 10 cycles of Ag deposition followed by 10 cycles of Au deposition on PAP/GCE surface.

**Fig. 5:**
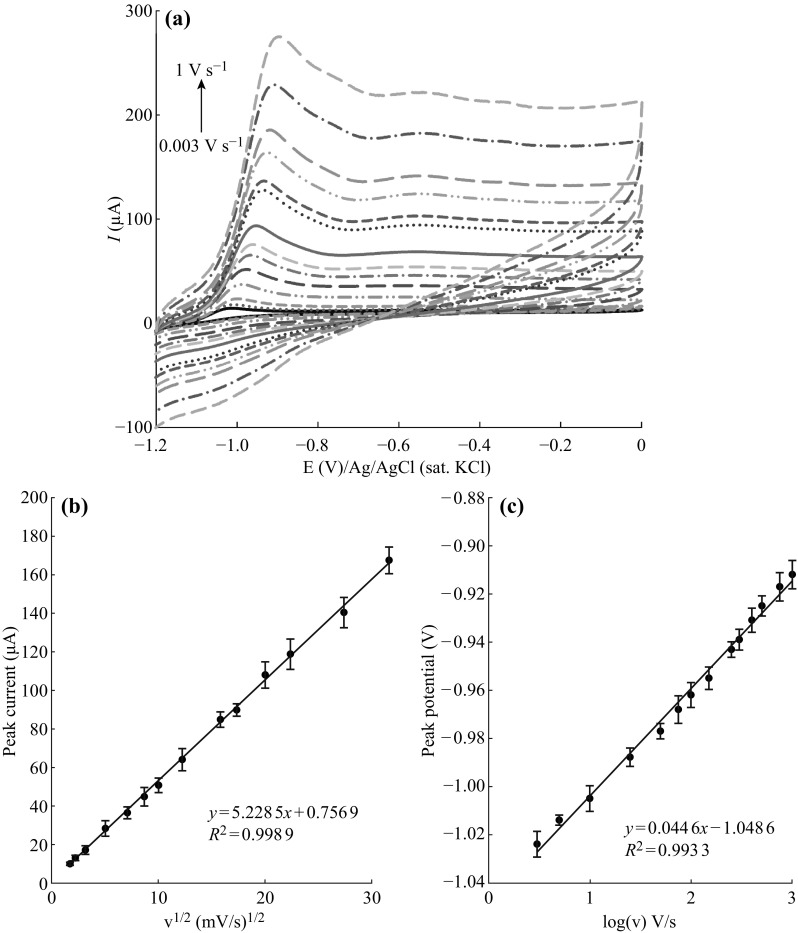
**a** CVs of Au–Ag/PAP/GC electrode in the presence of 1.0 mmol L^−1^ AB + 2 mol L^−1^NaOH with different scan rates of 0.003, 0.005, 0.010, 0.025, 0.050, 0.075, 0.100, 0.150, 0.250, 0.300, 0.400, 0.500, 0.750, and 1.000 V s^−1^. **b** *E*_p_ versus log*v* plot. **c** *I*_p_ versus *v* ^1/2^ plot

The effect of scan rate on the CV behavior of Au–Ag/PAP/GC electrode in the presence of 1.0 mM AB is shown in Fig. 5a. Cyclic voltammograms were obtained from potential scan between –1.2 to 0.0 V with different scan rates of 3–1000 mV s^−1^. The peak current increases linearly with the square root of scan rate over the range of 3–1000 mV s^−1^ (Fig. 5b). The linear regression equation was found as *i*_p_ (µA) = 3.076 *v* ^1/2^ ((mV s^−1^)^1/2^) + 1.6251 with a correlation coefficient of *R* ^2^ = 0.9994. According to these results, anodic peak current was controlled by the mass diffusion indicating a diffusion-controlled process. A plot of oxidation peak potential (*E*_*p*_) versus log*v* for Au–Ag/PAP/GCE is given in Fig. 5c. A linear relation between peak potential and logarithm of scan rate for AB oxidation peak indicates that there is an irreversible electrode process on Au–Ag/PAP/GCE surface. This is coherent with the linearity value that was calculated from the Laviron equation [58, 59].

**Fig. 6:**
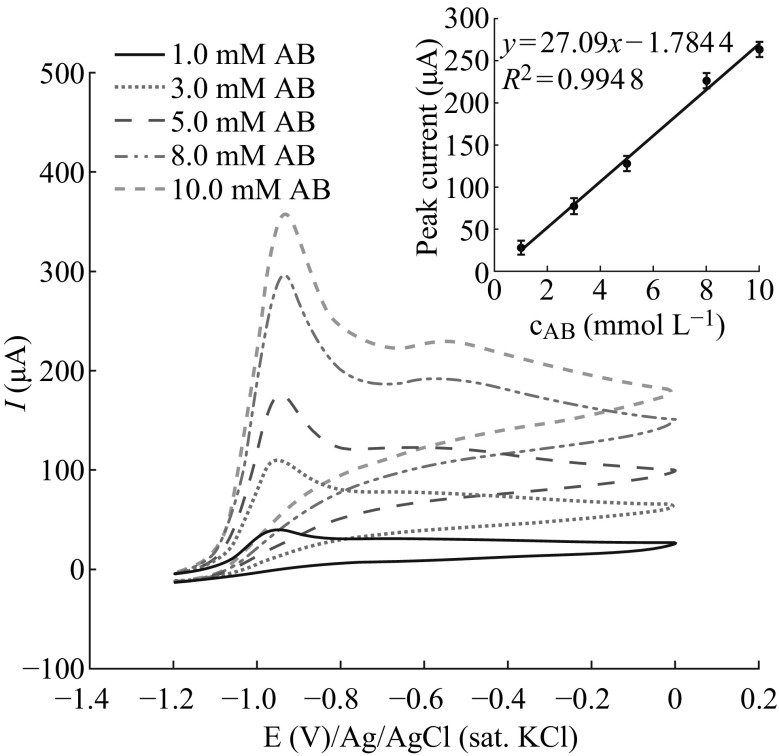
Cyclic voltammograms of Au–Ag/PAP/GC electrode in 2.0 mol L^−1^ NaOH presence with different AB concentrations where the scan rate is 0.050 V s^−1^. *Inset* Anodic peak current *vs*. AB concentration for the Au–Ag/PAP/GC electrode

The catalytic activity of Au–Ag/PAP/GCE was also studied in the concentration range between 1.0 and 10 mM of AB (Fig. 6). However, the oxidation peak potential of AB shifted slightly to more positive values with increasing concentrations of AB, and a linear relationship was observed between the peak current and AB concentration. Expectedly, for higher AB concentrations, it takes more time to consume all AB at the electrode surface where oxidation peak appears at more positive potentials. The linear dependence of peak current on AB concentrations indicates that Au–Ag/PAP/GC electrode shows well catalytic activity in all diluted or concentrated AB solutions in alkaline media.

In rotating disk electrode (RDE) voltammetry, the number of electrons involved in an oxidation reaction at a specific potential can be obtained from the Levich equation (Eq. 3) [60], where *n* is the number of the transferred electrons, *F* is the faraday constant (96,485 C mol^−1^), *A* is the electrode area (cm^2^), *D* is the diffusion coefficient (cm^2^ s^−1^), *ν* is the kinematic viscosity, and *C* is the analyte concentration (mol cm^−3^).

3 $$i = - 0.62nFAD^{2/3} v^{ - 1/6} C\omega^{1/2}.$$

**Fig. 7:**
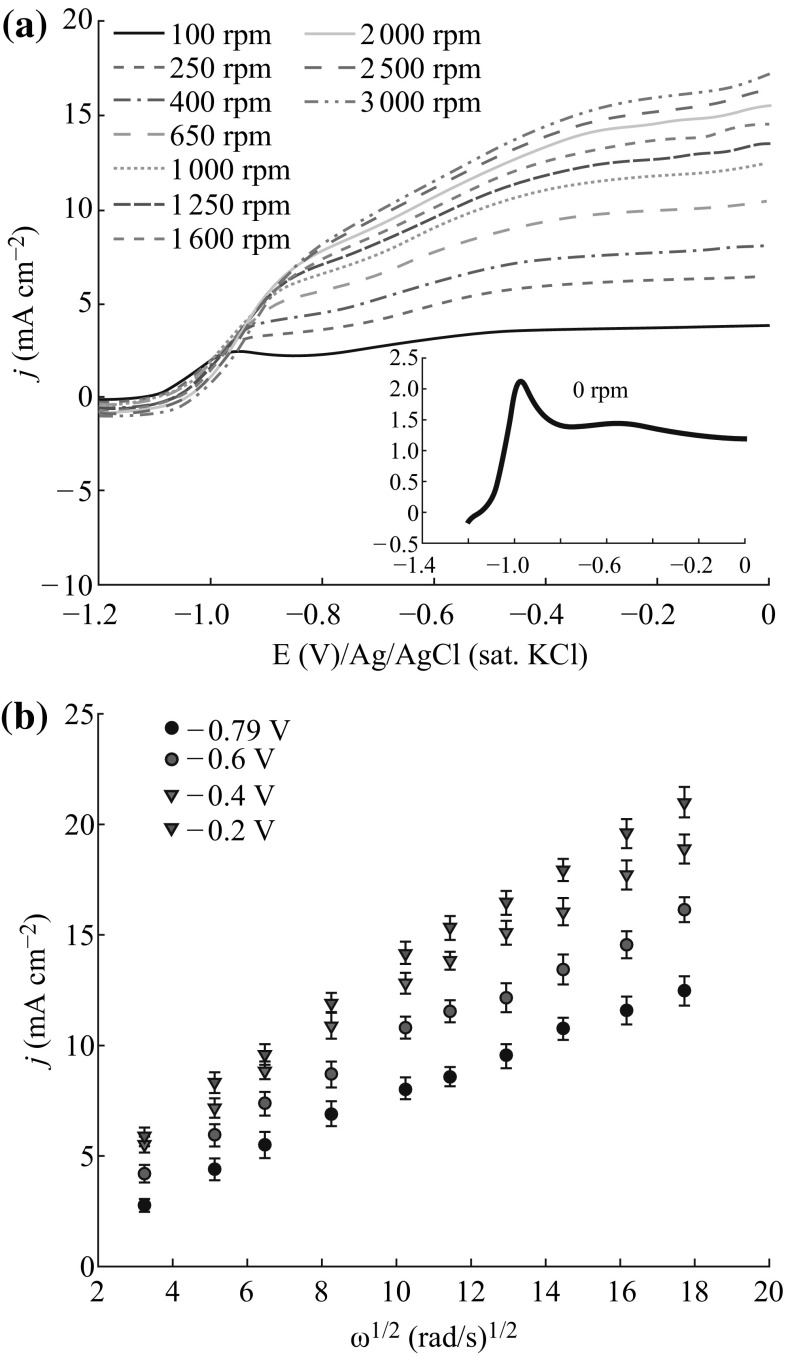
**a** LSVs of 5.0 mmol L^−1^ AB in 2.0 mol L^−1^ NaOH at Au–Ag/PAP/GCE rotated between 100 and 3000 rpm (the *inset* is 0 rpm). **b** Levich plots for AB oxidation at Au–Ag/PAP/GCE under the indicated potentials taken from **a**

In RDE experiment, *D* value for AB in 2.0 M NaOH media was taken as 8.45 × 10^−6^ cm^2^ s^−1^, which was determined by Nagle and Rohan [17]. The kinematic viscosity (*ν*) of solution was taken as 1.216 × 10^−2^ cm^2^ s^−1^ determined with Oswald viscosimeter at room temperature. A representative linear sweep voltammogram (LSV) of 5.0 mmol L^−1^ AB at Au–Ag/PAP/GCE is shown in the inset of Fig. 7a. In LSV study, AB oxidation peak was observed at −0.867 V on Au–Ag/PAP/GCE. In order to determine the number of electrons during AB oxidation, LSVs were obtained at Au–Ag/PAP/GCE with different rotation speeds (100–3000 rpm) (Fig. 7a). The oxidation currents increased with rotation rate indicate contribution of mass-transport control regime, at least within region of higher potentials. The curves also show the classical limiting current at higher potentials [61]. The large shift of half-wave potential to more positive values was observed as rotation speed of increased electrode. It has also been reported that Au indicates an irreversible reaction [62]. As expected, the complicated multielectron reactions involve formation of many intermediates on Au surface [63, 64]. The Levich graph represents the relationship between current density (*j*) and square root of rotation rate (*ω* ^1/2^) which draws various potentials as shown in Fig. 7b. According to the slope, the number of electrons transferred during the AB oxidation at Au–Ag/PAP/GCE is calculated to be 5.7 for high potential (−0.2 V). This value is close to the theoretical value of 6, as expected. The numbers of electrons transferred during the AB oxidation at Au disk electrode, Au/PAP/GCE, Ag/PAP/GCE, and Ag–Au/PAP/GCE were also calculated as 5.9, 6.0, 4.5, and 5.0, respectively.

**Fig. 8:**
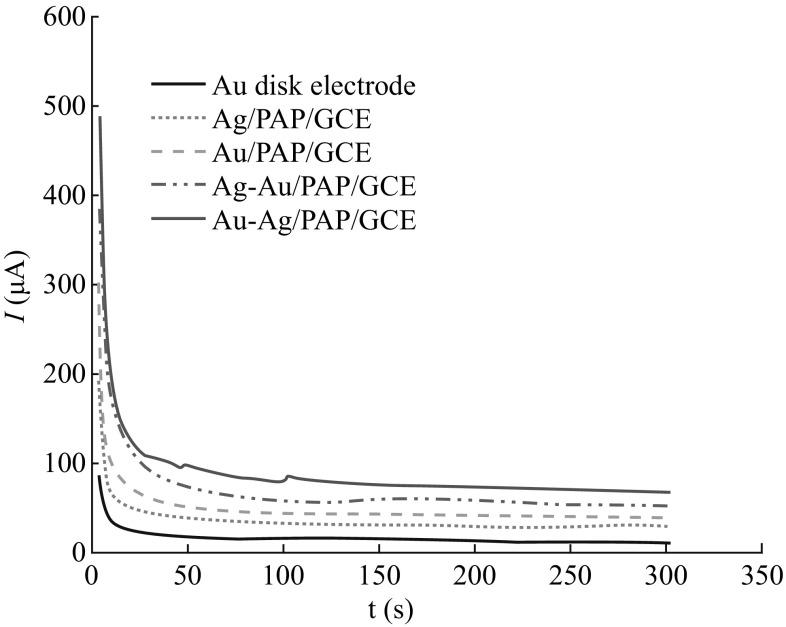
Chronoamperometric curves of 8.0 mmol L^−1^ AB oxidation on Au disk, Au/PAP/GCE, Ag/PAP/GCE, Ag–Au/PAP/GCE, and Au–Ag/PAP/GCE electrodes in 2.0 mol L^−1^ NaOH

The chronoamperometry measurements reveal more information about electrocatalytic activity and stability of the catalysts. The chronoamperometry responses of Au disk, Au/PAP, Ag/PAP, Ag–Au/PAP, and Au–Ag/PAP-modified GC electrodes in the presence of 8 mmol L^−1^ AB for 300 s are shown in Fig. 8. The current values of all modified electrodes decrease rapidly in the initial stage, and then reach steady-state characteristics. The highest steady-state peak current obtained at Au–Ag electrode corresponds to the high catalytic activity of this electrode for AB oxidation compared to other electrodes.

In order to estimate the number of electrons exchanged (*n*) of AB, the Cottrell equation was used based on chronoamperometry measurement [65]:

4 $$i = \frac{{nFACD^{1/2} }}{{\pi^{1/2} t^{1/2} }}.$$

Here *i* is the current density of electrodes (mA cm^−2^), *F* is the faradaic constant (96,485 C mol^−1^), *A* is the electrode area (cm^2^), *C* is the bulk borohydride concentration (mol cm^−3^), and *D* is the diffusion coefficient (cm^2^ s^−1^) taken as 8.45 × 10^−6^ cm^2^ s^−1^. The calculated *n* value from *i* versus *t* ^−1/2^ graph for Au disk electrode, Au/PAP, Ag/PAP, Ag–Au/PAP, and Au–Ag/PAP electrodes are 5.96, 6.04, 4.5, 5.3, and 5.8, respectively. These values are coherent with the electrons calculated with RDE.

## Conclusion

In the present study, well-dispersed Au/PAP, Ag/PAP, and Au–Ag bimentallic nanoparticles were successfully synthesized on polymer PAP/GCE films by electrochemical reduction process with CV technique. An average diameter of Au, Ag, and Au–Ag nanoparticles on the polymer films are 150, 80, and 85 nm, respectively, while the Au–Ag bimetallic nanoparticles were in the range of 20–50 nm. Au–Ag/PAP/GCE exhibits relatively high electrocatalytic activity for AB oxidation in terms of both oxidation peak potential and peak current. The highest catalytic activity may be attributed to the synergistic effect, unique structure, and homogenous dispersion of Au–Ag bimentallic nanoparticles on PAP/GCE surface. Additionally, Au–Ag/PAP/GCE exhibits high electrocatalytic activity for AB. This will be attractive to anode materials for ammonia borane fuel cells under alkaline conditions.

## References

[1] Henrietta WL, McGrady GS (2007). Non-hydride systems of the main group elements as hydrogen storage materials. Coordin. Chem. Rev..

[2] Metin Ö, Kayhan E, Özkar S, Schneider JJ (2012). Palladium nanoparticles supported on chemically derived graphene: an efficient and reusable catalyst for the dehydrogenation of ammonia borane. Int. J. Hydrogen Energy.

[3] Chiriac R, Toche F, Demirci UB, Miele P (2012). Ammonia borane thermolytic decomposition in the presence of metal (II) chlorides. Int. J. Hydrogen Energy.

[4] Rachiero GP, Demirci UB, Miele P (2011). Bimetallic RuCo and RuCu catalysts supported on γ-Al_2_O_3_. A comparative study of their activity in hydrolysis of ammonia-borane. Int. J. Hydrogen Energy.

[5] Liao J, Li H, Zhang X (2015). Preparation of Ti supported Co film composed of Co nanofibers as catalyst for the hydrolysis of ammonia borane. Catal. Commun..

[6] Gangal AC, Kale P, Edla R, Manna J, Sharma P (2012). Study of kinetics and thermal decomposition of ammonia borane in presence of silicon nanoparticles. Int. J. Hydrogen Energy.

[7] M.B.M. Conchaa, M. Chateneta, F.H.B. Limab, E.A. TicianelliI, In situ Fourier transform infrared spectroscopy and on-line differential electrochemical mass spectrometry study of the NH_3_BH_3_ oxidation reaction on gold electrodes. Electrochim. Acta **89**, 607–615 (2013). doi:10.1016/j.electacta.2012.11.027

[8] Rakap M, Özkar S (2010). Hydrogen generation from the hydrolysis of ammonia-borane using intra zeolite cobalt(0) nanoclusters catalyst. Int. J. Hydrogen Energy.

[9] Demirci UB, Miele P (2010). Hydrolysis of solid ammonia borane. J. Power Sources.

[10] Rakap M, Özkar S (2011). Hydroxyapatite-supported palladium(0) nanoclusters as effective and reusable catalyst for hydrogen generation from the hydrolysis of ammonia-borane. Int. J. Hydrogen Energy.

[11] Basu S, Brockman A, Gagare P, Zheng Y, Ramachandran PV, Delgass WN, Gore JP (2009). Chemical kinetics of Ru-catalyzed ammonia borane hydrolysis. J. Power Sources.

[12] Zahmakıran M, Ayvalı T, Akbayrak S, Çalışkan S, Çelik D, Özkar S (2011). Zeolite framework stabilized nickel(0) nanoparticles: active and long-lived catalyst for hydrogen generation from the hydrolysis of ammonia-borane and sodium borohydride. Catal. Today.

[13] Yang X, Cheng F, Tao Z, Chen J (2011). Hydrolytic dehydrogenation of ammonia borane catalyzed by carbon supported Co core-Pt shell nanoparticles. J. Power Sources.

[14] Wen M, Zhou S, Wu Q, Zhang J, Wu Q, Wang C, Sun Y (2013). Construction of NiCo-Pt nanopolyhedron inlay-structures and their highly efficient catalysis hydrolytic dehydrogenation toward ammonia borane. J. Power Sources.

[15] Kiran V, Kalidindi SB, Jagirdar BR, Sampath S (2011). Electrochemical oxidation of boron containing compounds on titanium carbide and its implications to direct fuel cell. Electrochim. Acta.

[16] Nagle LC, Rohan JF (2011). Nanoporous gold catalyst for direct ammonia borane fuel cells. J. Electrochem. Soc..

[17] Nagle LC, Rohan JF (2006). Ammonia borane oxidation at gold microelectrodes in alkaline solutions. J. Electrochem. Soc..

[18] Zhang XB, Han S, Yan JM, Shioyama H, Kuriyama N, Kobayashi T, Xu Q (2009). Electrochemical oxidation of ammonia borane on gold electrode. Int. J. Hydrogen Energy.

[19] Zhang XB, Yan JM, Han S, Shioyama H, Xu Q (2009). Magnetically recyclable Fe@Pt core-shell nanoparticles and their use as electrocatalysts for ammonia borane oxidation: the role of crystallinity of the core. J. Am. Chem. Soc..

[20] Mazloum-Ardakani M, Dehghani-Firouzabadi A, Sheikh-Mohseni MA, Benvidi A, Mirjalili BBF, Zare R (2015). A self-assembled monolayer on gold nanoparticles modified electrode for simultaneous determination of isoproterenol and uric acid. Measurement.

[21] Roustom BE, Siné G, Fóti G, Comninellis C (2007). A novel method for the preparation of bi-metallic (Pt–Au) nanoparticles on boron doped diamond (BDD) substrate: application to the oxygen reduction reaction. J. Appl. Electrochem..

[22] Duan D, Liang J, Liu H, You X, Wei H, Wei G, Liu S (2015). The effective carbon supported core–shell structure of Ni@Au catalysts for electro-oxidation of borohydride. Int. J. Hydrogen Energy.

[23] Atar N, Eren T, Yola ML, Karimi-Maleh H, Demirdogen B (2015). Magnetic iron oxide and iron oxide@gold nanoparticle anchored nitrogen and sulfur functionalized reduced graphene oxide electrocatalyst for methanol oxidation. RSC Adv..

[24] Azadbakht A, Abbasi AR, Derikvand Z, Karimi Z (2015). The electrochemical behavior of Au/AuNPs/PNA/ZnSe-QD/ACA electrode towards CySH oxidation. Nano-Micro Lett..

[25] Taei M, Hasanpour F, Salavati H, Banitaba SH, Kazemi F (2016). Simultaneous determination of cysteine, uric acid and tyrosine using Au-nanoparticles/poly(E)-4-(p-tolyldiazenyl)benzene-1,2,3-triol film modified glassy carbon electrode. Matter. Sci. Eng. C.

[26] Yola ML, Atar N (2014). A novel voltammetric sensor based on gold nanoparticles involved in *p*-aminothiophenol functionalized multi-walled carbon nanotubes: application to the simultaneous determination of quercetin and rutin. Electrochim. Acta.

[27] Atmeh M, Alcock-Earley BE (2011). A conducting polymer/Ag nanoparticle composite as a nitrate sensor. J. Appl. Electrochem..

[28] Chatenet M, Micoud F, Roche I, Chainet E (2006). Kinetics of sodium borohydride direct oxidation and oxygen reduction in sodium hydroxide electrolyte Part I. BH_4_-electro-oxidation on Au and Ag catalysts. Electrochim. Acta.

[29] Lu Y, Chen W (2012). Size effect of silver nanoclusters on their catalytic activity for oxygen electro-reduction. J. Power Sources.

[30] Yallappa S, Manjanna J, Dhananjaya BL, Vishwanatha U, Ravishankar B, Gururaj H, Niranjana P, Hungund BS (2016). Phytochemically functionalized Cu and Ag Nanoparticles embedded in MWCNTs for enhanced antimicrobial and anticancer properties. Nano-Micro Lett..

[31] Jiang Y, Gang J, Xu SY (2012). Contact mechanism of the Ag-doped trimolybdate nanowire as an antimicrobial agent. Nano-Micro Lett..

[32] Karabiberoğlu SU, Ayan EM, Dursun Z (2013). Electroanalysis of caffeic acid in red wine and investigation of thermodynamic parameters using an Ag nanoparticles modified poly (thiophene) film glassy carbon electrode. Electroanalysis.

[33] Juárez MF, Soldano G, Guesmi H, Tielens F, Santos E (2015). Catalytic properties of Au electrodes modified by an underlayer of Pd. Surf. Sci..

[34] Banerjee I, Kumaran V, Santhanam V (2015). Synthesis and characterization of Au@Pt nanoparticles with ultrathin platinum overlayers. J. Phys. Chem. C.

[35] Yi Q, Zhang J, Chen A, Liu X, Xu G, Zhou Z (2008). Activity of a novel titanium-supported bimetallic PtSn/Ti electrode for electrocatalytic oxidation of formic acid and methanol. J. Appl. Electrochem..

[36] Anjos DMD, Kokoh KB, LéGer JM, De Andrade AR, Olivi P, Tremiliosi-Filho G (2006). Electrocatalytic oxidation of ethanol on Pt-Mo bimetallic electrodes in acid medium. J. Appl. Electrochem..

[37] Ojani R, Raoof JB, Hasheminejad E (2013). One-step electroless deposition of Pd/Pt bimetallic microstructures by galvanic replacement on copper substrate and investigation of its performance for the hydrogen evolution reaction. Int. J. Hydrogen Energy.

[38] Mohl M, Dobo D, Kukovecz A, Konya Z, Kordas K, Wei J, Vajtai R, Ajayan PM (2011). Formation of CuPd and CuPt bimetallic nanotubes by galvanic replacement reaction. J. Phys. Chem. C.

[39] Ko FH, Tai MR, Liu FK, Chang YC (2015). Au–Ag core–shell nanoparticles with controllable shell thicknesses for the detection of adenosine by surface enhanced Raman scattering. Sens. Actuator B.

[40] Raoof JB, Ojani R, Rashid-Nadimi S (2010). Electrochemical synthesis of bimetallic Au@Pt nanoparticles supported on gold film electrode by means of self-assembled monolayer. J. Elecetroanal. Chem..

[41] Dursun Z, Ulubay Ş, Gelmez B, Ertas FN (2009). Electrocatalytic reduction of oxygen on a Pd ad-layer modified Au (111) electrode in alkaline solution. Catal. Lett..

[42] Jayakumar K, Rajesh R, Dharuman V, Venkatasan R, Hahn JH, Pandian SK (2012). Gold nano particle decorated graphene core first generation PAMAM dendrimer for label free electrochemical DNA hybridization sensing. Biosens. Bioelectron..

[43] Li Z, Fan GL, Tan ZQ, Li ZQ, Guo Q, Xiong DB, Zhang D (2016). A versatile method for uniform dispersion of nanocarbons in metal matrix based on electrostatic interactions. Nano-Micro Lett..

[44] Jayabal S, Ramaraj R (2014). Bimetallic Au/Ag nanorods embedded in functionalized silicate sol–gel matrix as an efficient catalyst for nitrobenzene reduction. Appl. Catal. A-Gen..

[45] Shi Q, Diao G, Mu S (2014). The electrocatalytic oxidation of glucose on the bimetallic Au–Ag particles-modified reduced graphene oxide electrodes in alkaline solutions. Electrochim. Acta.

[46] Neppolian B, Wang C, Ashokkumar M (2014). Sonochemically synthesized mono and bimetallic Au–Ag reduced graphene oxide based nanocomposites with enhanced catalytic activity. Ultrason. Sonochem..

[47] Yen C-W, Lin M-L, Wang A, Chen S-A, Chen J-M, Mou C-Y (2009). CO oxidation catalyzed by Au–Ag bimetallic nanoparticles supported in mesoporous silica. J. Phys. Chem. C.

[48] Tsai T-H, Thiagarajan S, Chen S-M (2010). Green synthesized Au–Ag bimetallic nanoparticles modified electrodes for the amperometric detection of hydrogen peroxide. J. Appl. Electrochem..

[49] Rahman L, Shah A, Khan SB, Asiri AM, Hussain H (2015). Synthesis, characterization, and application of Au–Ag alloy nanoparticles for the sensing of an environmental toxin, pyrene. J. Appl. Electrochem..

[50] Koçak ÇC, Dursun Z (2013). Simultaneous determination of ascorbic acid, epinephrine and uric acid at over-oxidized poly (*p*-aminophenol) film modified electrode. J. Electroanal. Chem..

[51] Bayler A, Schier A, Bowmaker GA, Schmidbaur H (1996). Gold is smaller than silver. crystal structures of [bis(trimesitylphosphine)gold(i)] and [bis(trimesitylphosphine)silver(i)] tetrafluoroborate. J. Am. Chem. Soc..

[52] Zheng D, Hu C, Gan T, Dang X, Hu S (2010). Preparation and application of a novel vanillin sensor based on biosynthesis of Au–Ag alloy nanoparticles. Sens. Actuator B.

[53] Bakır ÇC, Sahin N, Polat R, Dursun Z (2011). Electrocatalytic reduction of oxygen on bimetallic copper-gold nanoparticles- multiwalled carbon nanotube modified glassy carbon electrode in alkaline solution. J. Electroanal. Chem..

[54] Zhang X, Wei C, Song Y, Song X, Sun Z (2014). Nanoporous Ag-ZrO_2_ composites prepared by chemical dealloying for borohydride electrooxidation. Int. J. Hydrogen Energy.

[55] Zhao D, Yu Y, Xu C (2016). A sensitive electrochemical immunosensor for the detection of human chorionic gonadotropin based on a hierarchical nanoporous AuAg alloy. RSC Adv..

[56] Zhang Y, Gao G, Qian Q, Cui D (2012). Chloroplasts-mediated biosynthesis of nanoscale Au–Ag alloy for 2-butanone assay based on electrochemical sensor. Nanoscale Res. Lett..

[57] Wang AQ, Liu JH, Liu SD, Lin TS, Mou CY (2005). A novel efficient Au–Ag alloy catalyst system: preparation, activity, and characterization. J. Catal..

[58] Laviron E (1979). The use of linear potential sweep voltammetry and of A.C. voltammetry for the study of the surface electrochemical reaction of strongly adsorbed systems and redox modified electrodes. J. Electroanal. Chem..

[59] Guo W, Geng M, Zhou L, Chao S, Yang R, An H, Liu H, Cui C (2013). Multi-walled carbon nanotube modified electrode for sensitive determination of an anesthetic drug: tetracaine hydrochloride. Int. J. Electrochem. Sci..

[60] Bard AJ, Faulkner LR (2001). Electrochemical Methods: Fundamentals and Application.

[61] Lima FHB, Pasqualeti AM, Concha MBM, Chatenet M, Ticianelli EA (2012). Borohydride electrooxidation on Au and Pt electrodes. Electrochim. Acta.

[62] Cheng H, Scott K (2006). Determination of kinetic parameters for borohydride oxidation on a rotating Au disk electrode. Electrochim. Acta.

[63] Gyenge E (2004). Electrooxidation of borohydride on platinum and gold electrodes: implications for direct borohydride fuel cells. Electrochim. Acta.

[64] Mirkin MV, Yang H, Bard AJ (1992). Borohydride oxidation at a gold electrode. J. Electrochem. Soc..

[65] Wei JL, Wang XY, Wang Y, Chen QQ, Pei F, Wang YS (2009). Investigation of carbon-supported Au hollow nanospheres as electrocatalyst for electrooxidation of sodium borohydride. Int. J. Hydrogen Energy.

